# Long-term follow-up of nutritional status in children with GLUT1 Deficiency Syndrome treated with classic ketogenic diet: a 5-year prospective study

**DOI:** 10.3389/fnut.2023.1148960

**Published:** 2023-05-24

**Authors:** Ramona De Amicis, Alessandro Leone, Marta Pellizzari, Andrea Foppiani, Alberto Battezzati, Chiara Lessa, Anna Tagliabue, Cinzia Ferraris, Valentina De Giorgis, Sara Olivotto, Roberto Previtali, Pierangelo Veggiotti, Simona Bertoli

**Affiliations:** ^1^ICANS-DIS, Department of Food Environmental and Nutritional Sciences, University of Milan, Milan, Italy; ^2^Obesity Unit and Laboratory of Nutrition and Obesity Research, Department of Endocrine and Metabolic Diseases, IRCCS Istituto Auxologico Italiano, Milan, Italy; ^3^Clinical Nutrition Unit, Department of Endocrine and Metabolic Medicine, IRCCS Istituto Auxologico Italiano, Milan, Italy; ^4^Human Nutrition and Eating Disorder Centre, University of Pavia, Pavia, Italy; ^5^Ketogenic Metabolic Therapy Laboratory, Department of Public Health, Experimental and Forensic Medicine University of Pavia, Pavia, Italy; ^6^Department of Child Neurology and Psychiatry, IRCCS “C. Mondino” National Neurological Institute, Pavia, Italy; ^7^Pediatric Neurology Unit, “V. Buzzi” Hospital, Milan, Italy; ^8^Biomedical and Clinical Sciences Department, University of Milan, Milan, Italy

**Keywords:** GLUT1-Deficiency Syndrome, ketogenic diet, long-term effect, nutritional status, body composition, energy expenditure

## Abstract

**Introduction:**

The classic ketogenic diet (cKD) is an isocaloric, high fat, low-carbohydrate diet that induces the production of ketone bodies. High consumption of dietary fatty acids, particularly long-chain saturated fatty acids, could impair nutritional status and increase cardiovascular risk. The purpose of this study was to evaluate the long-term effects of a 5-year cKD on body composition, resting energy expenditure, and biochemical parameters in children affected by Glucose Transporter 1 Deficiency Syndrome (GLUT1DS).

**Methods:**

This was a prospective, multicenter, 5-year longitudinal study of children with GLUT1DS treated with a cKD. The primary outcome was to assess the change in nutritional status compared with pre-intervention, considering anthropometric measurements, body composition, resting energy expenditure, and biochemical parameters such as glucose and lipid profiles, liver enzymes, uric acid, creatinine, and ketonemia. Assessments were conducted at pre-intervention and every 12 months of cKD interventions.

**Results:**

Ketone bodies increased significantly in children and adolescents, and remained stable at 5 years, depending on the diet. No significant differences were reported in anthropometric and body composition standards, as well as in resting energy expenditure and biochemical parameters. Bone mineral density increased significantly over time according to increasing age. Body fat percentage significantly and gradually decreased in line with the increase in body weight and the consequent growth in lean mass. As expected, we observed a negative trend in respiratory quotient, while fasting insulin and insulin resistance were found to decrease significantly after cKD initiation.

**Conclusion:**

Long-term adherence to cKD showed a good safety profile on anthropometric measurements, body composition, resting energy expenditure, and biochemical parameters, and we found no evidence of potential adverse effects on the nutritional status of children and adolescents.

## 1. Introduction

Glucose Transporter 1 Deficiency Syndrome (GLUT1-DS; OMIM #606777) is a rare neurometabolic disorder resulting from an autosomal dominant mutation in SLCA1 (solute carrier family 2 member 1), a gene that encodes GLUT-1, the main transporter of glucose across the blood–brain barrier (BBB) and the plasma membrane of astrocytes. This genetic defect of GLUT-1 compromises the glucose uptake into the brain leading to an energy crisis. Patients with GLUT1-DS typically present with seizures, complex motor disorders, and impaired neurodevelopment (1, 2).

Since the initial description of GLUT1-DS in 1991 (3), the only known medical therapy for GLUT1-DS is the classic ketogenic diet (cKD), a normocaloric, hyperlipidic, normoprotein and low-carbohydrate diet, whose main purpose is to induce the constant production of ketone bodies, mainly acetoacetate (ACA) and β-hydroxybutyrate (BHB). This diet simulates the effects of long-term fasting, but does not deprive the body of the necessary calories for growth and development (1, 4). In GLUT1-DS, cKD is effective because the transport-mechanism for carbohydrates is inadequate and ketone bodies replaces carbohydrates as a source of energy for the brain (1). Moreover, other results suggest that this diet has an antiepileptic effect due to ketone bodies, that are involved in the alteration of mitochondrial function (e.g., promoting ATP synthesis and increasing mitochondrial biogenesis), in a decrease in glutamate release and its concentration in the synaptic cleft, in the activation of γ-aminobutyric acid (GABA) synthesis, and in the protection of the neurons against oxidative stress through various cellular mechanisms, such as the increase of reduced glutathione (GSH) and the increase of uncoupling protein (UCP) expression (5–10).

In the cKD, the intake of each macronutrient is designed according to the ketogenic ratio (KR), which is the ratio of the amount of fat to the sum of the amounts of carbohydrate and protein (both expressed in grams). The most common ratios used in clinical protocols are 4:1 and 3:1, i.e., 4–3 g of fat versus 1 g of protein and carbohydrate. The choice of KR depends on age, individual production, and brain sensitivity to ketone bodies (2–4): in clinical practice the KR may differ from 2,5–4:1 since the treatment with cKD in patients with GLUT1-DS is lifelong, so clinicians aim to reduce the amount of fat to a level without symptoms (seizures, paroxymal exercise-induced dyskinesia, lack of energy) and a level of ketosis within the recommended range. Given the high lipid content and the reduction in other nutrients (carbohydrates, fiber, vitamins, and minerals), short-term effects could include acidosis, hypoglycemia, dehydration, lethargy, and gastrointestinal symptoms such as constipation, nausea, and abdominal pain (11, 12). One study reported that only insulin resistance and insulin sensitivity indexes changed after 12 weeks of cKD, suggesting that KD seems to have no effect on inflammatory cytokines production and abdominal fat distribution in the short term (2). Tagliabue et al. found no statistically significant differences in the gut microbiota at 3 months, except for a bacterial group suggested to be involved in the exacerbation of the inflammatory state of the intestinal mucosa associated with animal fat consumption (13). Administering a 6-month cKD to patients with medically refractory epilepsy, Tagliabue et al. found an increase in fat oxidation and a decrease in respiratory quotient, without appreciable changes in resting energy expenditure (REE) (14). In addition, the possible effects of high fat consumption might include vitamin deficiencies, hyperlipidemia, kidney stones, reduced bone mineral density, weight loss, and reduced height gain (15). However, only a few studies have investigated the long-term effects of cKD (16, 17). A ten-year study of a sample of 10 children showed that initial dyslipidemia normalized and no significant difference was observed in Body Mass Index (BMI), systolic and diastolic blood pressure, as well as carotid intima-media thickness (17). Regarding nutritional status, which includes growth pattern, body composition, bone mineral density, biochemical parameters, and energy expenditure (18), one study revealed that most GLUT1-DS patients (80%) maintained or even improved their growth pattern at 12-month follow-up (19). Other studies have shown that after 12 months of cKD there were no significant changes in ghrelin and leptin, nor in body fat, glucose and lipid profile (16, 20). Only in one study was cKD administered for more than 5 years. This study, however, was exclusively focused on body composition, bone mineral content, and bone mineral density, and it was conducted on a case series of GLUT1-DS adults, suggesting that maintenance of a cKD does not result in major adverse effects (21). However, no study investigated the long-term effects of a cKD on all nutritional status components in children.

Therefore, the aim of our study was to evaluate the long-term effects of a 5-year cKD on body composition, resting energy expenditure, and biochemical parameters in a sample of children affected by GLUT1-DS.

## 2. Methods

### 2.1. Ethics statement

The study protocol was approved by the ethics committee of the Fondazione IRCCS Policlinico San Matteo di Pavia (reference number 20180083746) and complied with all principles of the Declaration of Helsinki. All caregivers provided written informed consent before the beginning of the study.

### 2.2. Study design and inclusion criteria

This was a prospective, multicenter, 5-year longitudinal study of children with GLUT1DS treated with a cKD. The primary outcome was to evaluate the change from pre-intervention in nutritional status, including anthropometric measurements, body composition, resting energy expenditure, and biochemical parameters, such as glucose, insulin, HbA1c, HOMA-IR, lipid profile (triglycerides [TGs], total cholesterol [TC], low-density lipoprotein cholesterol [LDL-C], and high-density lipoprotein cholesterol [HDL-C]), liver enzymes (alanine aminotransferase[ALT], aspartate aminotransferase[AST], gamma-glutamyl transferase [GGT]), uric acid, creatinine, and ketonemia.

We conducted assessments prior to the initiation of cKD intervention and then every 12 months or 5 years during the cKD interventions.

### 2.3. Participants

Patients were recruited from the Department of Child Neuropsychiatry of the Casimiro Mondino IRCCS Foundation (Pavia, Italy) and the Pediatric Neurology Unit of the Vittore Buzzi Hospital (Milan, Italy) starting in October 2010 and followed until March 2019.

Patients were required to have no contraindications as defined by the latest Consensus (4), such as β-oxidation defects, carnitine deficiency, pyruvate carboxylase deficiency, porphyria and other specific disorders involving fatty acid transport and oxidation. Moreover, we excluded children with parents or caregivers that were noncompliant or unable to maintain adequate nutrition (4).

Nutritional measurements were performed at the International Center for the Assessment of Nutritional Status (ICANS) of the University of Milan. cKDs were implemented at the Center for Research on Human Nutrition and Eating Disorders in Pavia and at ICANS of the University of Milan according to similar guidelines (4).

Fifteen children and adolescents (11 females and 4 males, mean age 8.6 ± 3.0 years), all diagnosed with GLUT1-DS, were prospectively enrolled. Specifically, patients with GLUT1- DS underwent fasting lumbar puncture (at least 5–6 h of fasting), where a cerebrospinal fluidglucose level less than 0.6 was considered suspicious for the presence of GLUT1-DS (22). In addition, for final confirmation, all patients were subjected to mutation analysis of the SLC2A1 gene.

### 2.4. Ketogenic diet setting

Patients started the dietary protocol at home, with a gradual increase in the ketogenic ratio and with no fasting required, according to the previously published protocol (2).

At pre-intervention, each patient filled out a dietary history with a registered dietician to assess their habitual caloric intake, intolerances, and food preferences. The cKD was tailored to the patient, considering REE-related energy expenditure measured by indirect calorimetry and physical activity level, as well as the appropriate ketogenic ratio to be achieved. Where necessary, appropriate modifications were made to the caloric prescriptions during follow-up. All participants received a normocaloric diet, with a minimum protein intake of 0.7 g/kg (23) and a sugar-free, multivitamin, multimineral, and potassium citrate supplementation (depending on gender and age). Each patient’s allergies, intolerances, and food preferences were also taken into account. Before the beginning of the dietary protocol, patients and caregivers received preliminary counseling to clarify any doubts and facilitate understanding of the ketogenic diet, the attention and time required for meal preparation, food costs, and possible side effects of the diet.

Table 1 shows macronutrient composition and ketogenic ratio at the beginning of the cKD after the first week of ketosis induction.

**Table 1:** Composition of the prescribed cKDs.

	Mean	SD
Energy intake (kcal/day)	1,532	535
Energy intake/BW (kcal/kg)	46.1	23.0
Protein (g/day)	29.9	13.5
Protein/BW (g/kg)	0.9	0.6
Fat (g/day)	146.6	52.4
Fat (%)	86.3	10.1
SFA (g/day)	53.9	20.0
SFA (%)	36.1	11.7
Carbohydrate (g/day)	22.3	10.5
Carbohydrate (%)	5.8	2.7
Ketogenic ratio	2.9	0.6

BW, body weight; SFA, Saturated Fatty acids.

Patients started the diet therapy at home with an initial ratio of 1:1, then gradually increased to a ratio of 2:1, and finally 3:1 or 4:1. The final ketogenic ratio was determined based on patient tolerance and ketonemia trends to ensure stable blood BHB values >2.0 mmol/L.

To monitor patients during follow-up, caregivers were instructed to check and note capillary ketonemia and ketonuria daily.

### 2.5. Neurological assessment

Neurological evaluations and electroencephalography (EEG) were performed at pre-intervention and once a year thereafter, at the Department of Neurology and Child Psychiatry, Fondazione IRCCS Istituto Neurologico Casimiro Mondino in Pavia and at the Pediatric Neurology Unit, “V. Buzzi” Hospital in Milan, according to the 2011 Italian consensus on ketogenic therapy based on the WHO guidelines (22).

The following neurological symptoms were monitored: seizure types and their frequency, paroxysmal dyskinesia, spasticity, ataxia, dystonia, dysarthria, and muscle strength. Also, caregivers were asked to note alertness, activity, and seizure episodes on a daily basis.

### 2.6. Main outcomes: assessment of nutritional status

Nutritional status assessment is the result of anthropometric measurements, body composition in terms of fat and fat free mass, resting energy expenditure, and biochemical parameters (24).

*Anthropometric measurements* were performed by the same trained dietitian according to conventional measurement criteria and procedures (24).

Body weight (BW, kg) and body height (BH, cm) were measured with an accuracy of 100 g and 0.5 cm, respectively. Body mass index (BMI) was calculated using the formula Body Weight (kg) / Body Height^2^ (m^2^).

Sex-specific BMI-for-age percentiles and Z scores were calculated based on the 2000 Centers for Disease Control and Prevention (CDC) growth charts (25). According to CDC guidelines, a z-score of ≤ − 2 was considered severely underweight, a score between −2 and − 1 was considered underweight, between −1 and + 1 was considered normal weight, between +1 and + 2 was considered overweight, and a score ≥ 2 was considered obese.

Waist circumference (WC) was measured to the nearest 0.1 cm with a non-elastic tape. With the patient standing, and following normal exhalation, the measurement was taken at the midpoint between the last rib and the iliac crest in a horizontal plane. WC was quantified according to reference tables for age and sex (26).

Skinfold thickness was measured on the non-dominant side of the body using a Holtain LTD caliper at the triceps skinfold landmark. All measurements were taken in triplicate for all sites, and the mean of the three values was calculated. The intra-observer variation for skinfold measurements ranged from 2.5 to 2.9%.

Arm Muscular Area (AMA) and Arm Fat Area (AFA), indicators of nutritional status degree, muscle and fat mass amount, respectively, were calculated according to the following formulas:

AMA = [Arm circumference (cm) - (Tricipital Skinfold (mm) * 3.14)]^2^/ (4 * 3.14).

AFA = [Arm circumference (cm) / (4 * 3.14)] - AMA^2^.

AMA and AFA have been interpreted according to the CDC percentiles (25).

*Body composition assessment* was performed by Dual Energy X-Ray Absorptiometry (DEXA) at the ICANS center, using a GE Lunar iDXA, Boston, United States.

DEXA is a body composition analysis technique that allows the identification of three compartments: Bone Mineral Content (BMC), Lean Body Mass (LM), and Fat Mass (FM). Total body scans were performed by a single operator on all subjects in the supine position. The whole body of each subject was scanned in an average exposure time of 15 min. Coefficient of Variation were less than 1% for all measurements (27). Bone Mineral Density (BMD) can be obtained from this examination: a z-score < −2 indicates a value below the expected range for age, thus detecting presence of osteoporosis (27). We calculated fat mass index (FMI, kg/m2) in children and adolescents by dividing FM by the square of height. Total FM, Fat Mass Index, and Lean Mass Index were interpreted according to the body composition of reference children (28).

*Resting energy expenditure* (REE) was measured by means of an open-circuit ventilated hood system (Sensor Medics 29, Anaheim, CA, United States). All measurements were taken in the fasting state (minimum 12–14 h of fasting), for at least 30 min according to a detailed previously published protocol (14). REE was calculated using the abbreviated Weir equation (29).

Regarding *biochemical parameters*, fasting blood samples were collected by venipuncture of the antecubital vein in a sitting or reclining position, using vacuum-sealed test tubes. After centrifugation (800 g for 10 min at 5°C), aliquots of serum sample were stored at 80°C until further analysis. An autoanalyzer (Cobas Integra 400 plus, Roche Diagnostics, Mannheim) was used to determine serum concentrations of glucose, TC, HDL-C, LDL-C, TG, AST, ALT, GGT, creatinine, and uric acid. Circulating insulin was measured in duplicate by an autoanalyzer (Cobas e411 Hitachi, Roche Diagnostics). The homeostatic model of insulin resistance assessment (HOMA-IR) was calculated as [fasting glucose (mg/dL) × fasting insulin (mU/L)/405] (30). Glycated hemoglobin (HbA1c) was determined by turbidimetric inhibition immunoassay for hemolyzed whole blood using an autoanalyzer. The % HbA1c was obtained by the ratio of HbA1c concentration to total blood hemoglobin concentration. The following values were defined as high: TC ≥ 200 mg/dL, LDL-C > 130 mg/dL, TG > 150 mg/dL, HOMA-IR ≥ 3.16 for children, blood glucose ≥100 mg/dL, HbA1c ≥ 6%, and insulin >23 μU/mL, while the following values were considered low: HDL < 40 mg/dL for males and < 50 mg/dL for females (30–32). As for liver enzymes, AST ≥ 30 U/L, ALT ≥35 U/L, and GGT ≥ 18 U/L were considered high in females, while AST ≥ 45 U/L, ALT ≥40 U/L, and GGT ≥ 28 U/L were regarded as high in males according to the of the ICANS laboratory normal upper limit. Capillary ketonemia was measured with an *in vitro* β-ketone self-testing medical diagnostic device (GlucoMen LX PLUS, Menarini Diagnostics, test range 0.1 mmol/L-8.0 mmol/L). Ketonuria was measured using a urine ketone test (Ketostix®, Bayer Diabetes, Berkshire, United Kingdom).

### 2.7. Statistical analysis

Continuous variables are presented as mean ± standard deviation. An independent t-test was used to compare the means of nutritional and biochemical variables among GLUT1-DS children. Levene’s test was performed to assess the equality of variances for a variable calculated for the groups. A one-way repeated measures ANOVA with post-hoc Bonferroni comparison test was run to determine if there were differences in the variables of interest during the 5 years of dietary treatment.

## 3. Results

### 3.1. Pre-intervention

Table 2 shows the clinical characteristics of all patients, with mutations’ types, phenotypes and pharmacological treatments at diagnosis.

**Table 2:** Clinical characteristics of recruited patients.

Patient	Age at diagnosis	SLC2 A1 testing	Mutations	Phenotype	Pharmacological treatments	Kind of diet and KR	Age at the diet-start
1	6 years	Yes	Protein mutation c.26C > T, gene p.Thr9Met, p.T9M	Generalized epilepsy (absence seizures)	No	cKD - 3:1	6 years
2	9 years	Yes	Protein mutation R223W, gene p.Arg223Trp	Focal and generalized (absence epilepsy),PED (chorea, dystonia)	Oxcarbazepine	cKD - 2,5:1	9 years
3	5 years	Yes	Protein mutation R153C, gene p.Arg153Cys	Generalized epilepsy (absence epilepsy and tonic clonic sezures), chorea, ataxia, stroke like episodes	No	cKD - 2.5:1	5 years
4	9 years	Yes	Protein mutation R458W, gene p.Pro485Leu	Generalized epilepsy (tonic clonic sezures), PED (chorea and dystonia), migraine, weakness	Valproate	cKD - 2.5:1	9 years
5	10 years	Yes	Protein mutation g.33411C > T, gene p.Arg126Cys	Generalized epilepsy (tonic clonic sezures, myclonic absence sezures), PED (dystonia), intellectual disability	Levetiracetam	cKD - 2.5:1	11 years
6	9 years	Yes	Protein mutation V165I, gene p.Val165Ile	PED (chorea, dystonia), intellectual disability	No	cKD - 3:1	9 years
7	NA	Yes	Protein mutation R249Afs131X, gene p.Arg249Ala fs*131	Generalized epilepsy (myclonic absence seizures), intellectual disabilityi	No	cKD - 3:1	NA
8	15 years	Yes	Protein mutation R400H, gene p.Arg400His	Intellectual disability, PED (dystonia, myoclonias), ocular movement disorder	Valproate	cKD - 2.5:1	15 years
9	NA	Yes	Protein mutation c.370delC, gene p.Leu124Trpfsx12	Intellectual disability,generalized epilepsy (myclonic seizures), chorea and ataxia, ocular movement disorder	No	cKD - 3:1	NA
10	7 years	Yes	Protein mutation p.N34S, gene p.Asn34Ser	Intellectual disability, generalized epilepsy (absence seizures)	No	cKD - 3:1	7 years
11	7 years	Yes	Protein mutation C.1198 > T, gene p.Arg400Cys	Intellectual disability, generalized epilepsy (absence and myoclonic seizures), PED (dystonia) ocular movement disorder	No	cKD - 3:1	7 years
12	NA	Yes	Protein mutation c.884C > T, gene Thr295Met	Generalized epilepsy (absence seizures), PED (dystonia)	No	cKD - 2,5:1	3 years
13	NA	Yes	Protein mutation p.R223W	Generalized epilepsy (myoclonic seizures), ocular movement disorder	No	cKD - 2,5:1	4 years
14	NA	Yes	Protein mutation R458W, gene p.Pro485Leu	Generalized epilepsy (tonic clonic sezures), PED (chorea and dystonia), weakness	Valproate	cKD - 3:1	8 years
15	11 years	Yes	Protein mutation c.26C > T, gene p.Thr9Met, p.T9M	Generalized epilepsy (absence seizures)	No	cKD - 3:1	11 years

cKD, classic Ketogenic Diet; NA, not applicable; KR, ketogenic ratio; PED, Paroxysmal Exercise-induced Dyskinesia.

Fifteen patients were recruited. All patients resulted positive to the SLC2 A1 test. The main phenotypes were generalized epilepsy and intellectual disability. Ten patients (67%) were not following any drug therapy. Three patients started cKD being younger than 6 years old.

Table 3 shows the nutritional characteristics at baseline. Only two patients were underweight and one was obese, as shown also by high waist circumference, high body fat and high AFA. The remaining reported normal nutritional status and body composition. All 15 were included in the study.

**Table 3:** Nutritional characteristics at baseline.

	*N* (%)
Weight	
< −2 z-score	2 (13,3%)
> +2 z-score	1 (6,7%)
Height	
< −2 z-score	2 (13,3%)
> +2 z-score	0 (0%)
BMI	
< −2 z-score	2 (13,3%)
> +2 z-score	1 (6,7%)
Waist circumference	
< −2 z-score	0 (0%)
> +2 z-score	1 (6,7%)
Arm Circumference z-score	
< −2 z-score	1 (6,7%)
> +2 z-score	1 (6,7%)
Arm Muscle Area z-score	
< −2 z-score	1 (6,7%)
> +2 z-score	0 (0%)
Arm Fat Area z-score	
< −2 z-score	0 (0%)
> +2 z-score	1 (6,7%)

BMI, Body Mass Index, kg/m^2^.

### 3.2. Intervention

All patients completed the 5-year protocol. The range of the prescribed KRs was 2–3:1 with carbohydrates intake lower than 10% and fat percentage amounting to minimum 80%. Energy and protein intake was adjusted according to body weight (23), maintaining the same ketogenic ratio.

### 3.3. Post-intervention

All children reached the therapeutic range of KBs (beta-hydroxybutyrate >2.0 mmL/l) and tolerated the diet well.

**Figure 1:**
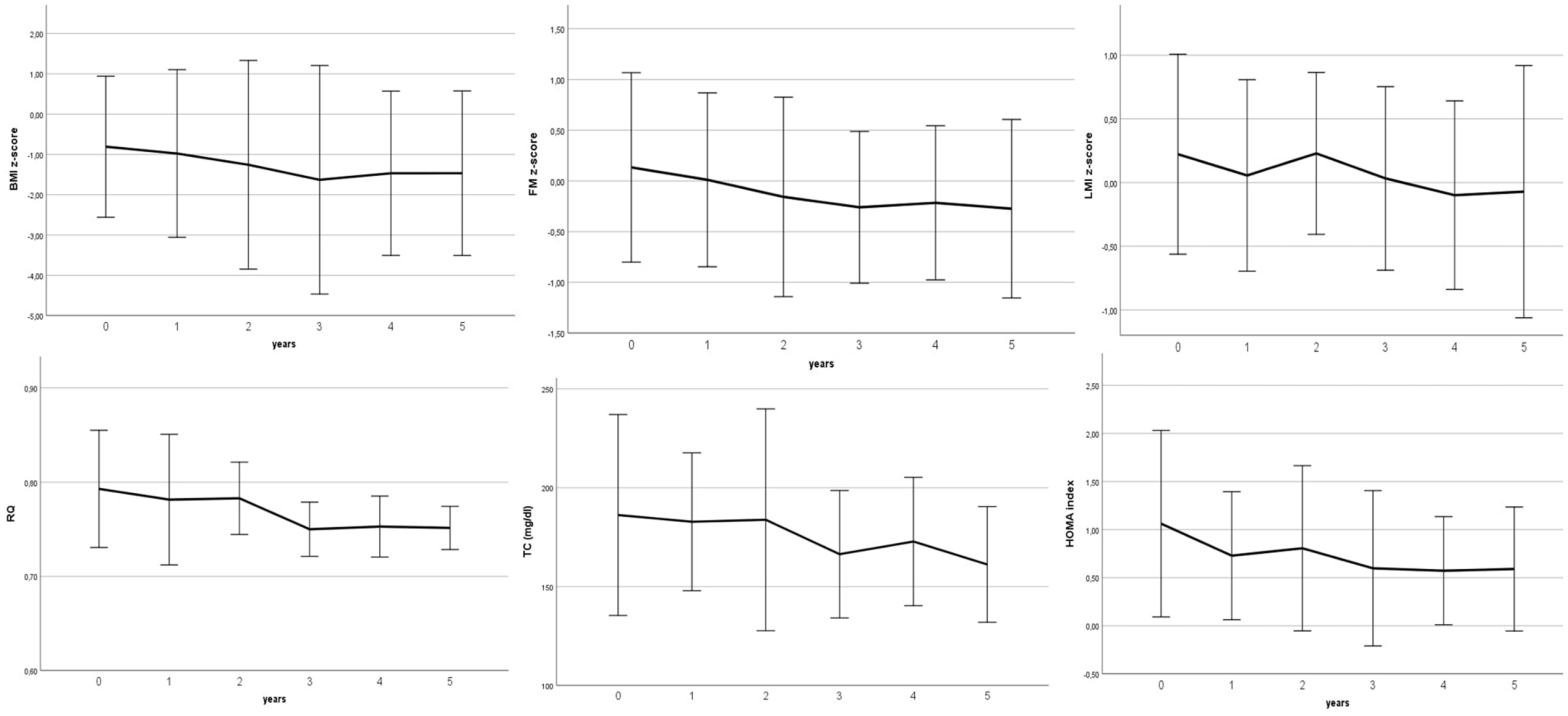
Timecourse of principal nutritional outcomes.

Table 4 and Figure 1 show the annual changes from the beginning of the cKD.

**Table 4:** Timecourse of the nutritional changes.

	Baseline		1 year		2 years		3 years		4 years		5 years		*p*-value
	Mean	SD	Mean	SD	Mean	SD	Mean	SD	Mean	SD	Mean	SD	
Anthropometric measurements													
Weight (kg)	33,2a	23.2	32,1a	19.3	33,9a	18.9	*38,6b*	19.4	*40,4b*	21.0	*40,9b*	19.1	**0.026**
Weight *z*-score	−0.5	2.0	−0.8	2.0	−0.9	2.1	−0.7	2.0	−0.9	2.0	−0.8	2.1	0.975
Height (cm)	124,9a	28.7	*128,6b*	26.2	*132,4b*	24.8	*137,7b*	26.3	*140,8b*	25.0	*142,0b*	22.0	**0.002**
Height *z*-score	−0.7	1.3	−0.8	1.1	−0.7	1.1	−0.6	1.1	−0.6	1.1	−0.7	1.1	0.994
BMI (kg/m^2^)	18.6	6.0	17.6	4.4	17.7	3.9	18.8	3.6	18.8	4.5	19.2	4.9	0.582
BMI z-score	−0.1	1.9	−0.6	2.1	−0.8	2.4	−0.8	3.1	−0.8	2.3	−0.8	2.4	0.978
Waist Circumference (cm)	61.9	19.2	60.9	14.6	62.7	13.4	66.2	13.0	66.5	13.5	66.8	13.2	0.546
Waist Circumference *z*-score	1.8	2.5	1.2	1.9	0.8	1.6	0.8	1.6	0.9	1.8	0.6	2.0	0.466
Arm Circumference (cm)	21.9	6.5	20.7	5.6	20.9	4.9	23.0	4.9	21.4	5.0	23.1	5.6	0.258
Arm Circumference *z*-score	0.1	0.6	−0.7	1.6	−0.7	1.2	−0.6	1.2	−0.7	1.1	−0.6	1.3	0.316
Triceps_skf (mm)	14.2	7.1	13.3	5.0	13.3	5.8	13.1	5.2	14.5	7.8	14.2	7.5	0.848
Arm Muscle Area (cm^2^)	25.5	14.6	23.3	13.5	23.1	9.9	28.6	11.2	27.8	13.5	30.1	15.7	0.216
Arm Muscle Area *z*-score	0.2	1.5	−0.7	1.6	−0.6	1.1	−0.5	1.0	−0.6	1.6	−0.7	1.5	0.516
Arm Fat Area (cm^2^)	15.8	10.4	13.1	7.7	13.6	8.4	15.3	8.0	10.5	6.6	14.6	10.7	0.313
Arm Fat Area *z*-score	0.4	1.4	0.0	0.7	−0.4	0.7	0.6	0.8	−0.3	0.8	−0.1	1.4	0.412
Body composition													
BMC (g)	1,056,7a	709.7	945,4a	474.2	1,186,6a	739.8	*1,567,4b*	844.2	*1,340,8b*	635.0	*1,470,1b*	898.6	**0.031**
BMD	0,780a	0.218	0,732a	0.151	0,810a	0.186	*0,898b*	0.205	*0,846b*	0.172	*0,925b*	0.211	**0.001**
BMD *z*-scores	0.1	0.7	0.1	1.0	−0.2	1.1	0.1	0.1	−0.1	1.1	−0.1	1.6	0.427
FM (kg)	10.8	8.8	8.1	5.5	10.2	7.4	13.3	6.2	10.7	6.7	12.2	9.1	0.284
FMI	5.9	3.0	4.9	1.8	5.4	2.3	6.1	1.8	5.1	2.2	5.4	3.5	0.100
FMI *z*-score	0.1	1.6	−0.5	1.1	−0.2	1.4	0.4	0.6	−0.6	1.2	−0.4	1.3	0.616
FM (%)	31.1	8.1	29.9	5.8	29.7	6.7	30.4	6.0	27,74b	5.9	27,43b	8.7	**0.013**
FM *z*-score	−0.1	1.7	−0.5	1.2	−0.4	1.6	0.3	0.6	−0.5	1.5	−0.3	1.3	0.077
LM (kg)	21,2a	14.5	20,9a	12.3	22,3a	12.7	24,6a	12.3	27,4b	14.0	30,7b	13.9	**0.001**
LMI	11,6a	2.0	11,3a	1.1	11,7a	2.1	13,1a	2.0	14,0b	2.3	14,3b	3.3	**0.032**
LMI *z*-score	0.2	1.2	0.1	1.2	−0.1	1.1	0.4	0.5	−0.1	1.2	−0.1	1.6	0.637
Resting energy expenditure													
VO_2_	0.1	0.1	0.2	0.1	0.1	0.1	0.2	0.1	0.2	0.0	0.2	0.0	0.543
VCO_2_	0.1	0.1	0.1	0.0	0.1	0.0	0.1	0.0	0.1	0.0	0.1	0.0	0.686
RQ	0,81a	0.1	0,77a	0.1	0,77a	0.0	*0,76b*	0.1	*0,76b*	0.0	*0,76b*	0.0	**0.041**
REE (kcal/die)	844a	473	896a	387	915a	392	*1059b*	399	*1153b*	333	*1146b*	374	**0.030**
REE/weight (kcal/kg weight)	36.0	10.7	39.1	10.9	39.7	11.3	33.1	13.1	34.9	7.6	31.7	9.7	0.260
Biochemical parameters													
Urea (mg/dl)	19	9	19	5	19	6	21	5	18	5	18	5	0.908
Creatinine (mg/dl)	0.3	0.1	0.3	0.1	0.3	0.1	0.4	0.1	0.4	0.1	0.4	0.1	0.146
Uric acid (mg/dl)	4.7	1.3	5.6	1.6	5.6	1.2	5.3	1.1	5.3	1.0	5.4	1.0	0.154
TC (mg/dl)	168	42	168	39	163	39	157	30	160	32	163	26	0.446
HDL-C (mg/dl)	56	15	59	18	57	16	56	16	52	14	51	11	0.978
LDL-C (mg/dl)	101	30	100	26	100	28	96	22	98	25	104	20	0.419
TC/HDL	3.1	0.7	3.0	0.7	3.0	0.7	2.9	0.6	3.3	1.0	3.3	0.6	0.549
LDL/HDL	1.9	0.6	1.8	0.5	1.9	0.7	1.8	0.5	2.0	0.8	2.1	0.5	0.997
TG (mg/dl)	66	24	58	18	57	17	64	24	66	25	64	29	0.525
Glucose (mg/dl)	87	5	79	10	79	8	86	7	86	9	87	11	0.108
Insulin (mU/mL)	10,9a	12.0	*4,5b*	2.9	*5,5b*	3.2	*5,8b*	6.5	*5,1b*	3.2	*5,7b*	2.9	**0.047**
HbA1c (%)	4.8	0.4	4.5	0.3	4.5	0.6	4.7	0.3	4.7	0.3	4.5	0.6	0.216
HOMA index	2,3a	2.6	*0,9b*	0.6	*1,0b*	0.8	*1,7b*	1.5	*1,3b*	0.7	*1,3b*	0.6	**0.048**
AST	29.1	15.4	26.4	7.0	25.3	8.0	21.2	6.5	22.4	9.7	20.0	6.3	0.290
ALT	20.5	23.9	22.2	10.1	19.2	12.8	13.9	4.1	16.5	5.6	14.9	7.8	0.649
GGT	14.5	8.1	12.9	6.0	11.2	5.2	11.6	4.5	11.3	3.6	10.7	4.2	0.340
KBs (mmol/L)	0.1	0.1	2.8	0.7	2.5	1.8	2.4	1.9	2.5	1.8	2.6	1.4	**0.041**

In bold, *p* < 0,05. a,b.

BMI, Body Mass Index; BMC, Bone Mineral Content; BMD, Bone Mineral Density; FM, Fat Mass; FMI, Fat Mass Index; LM, Lean Mass; LMI, Lean Mass Index, VO_2_, oxygen volume; VCO_2_, Carbon Dioxide Volume; RQ, Respiratory Quotient; REE, Resting Energy Expenditure; TC, Total Cholesterol; HDL, High Density Lipoprotein; LDL, Low Density Lipoprotein; TG, Tryglicerides; HBA1C, Glycosilated Hemoglobin; HOMA, Homeostasis model assessment; ALT, Alanine Amino Transferase; AST, Aspartate Amino Transferase; GGT, γ-glutamyl transferase; KB, Ketone Bodies.

KBs increased significantly in children and adolescents with values greater than 2 mmol/L, and remained stable at 5-y.

Concerning growth, we found a significant increase in weight and height during the time course but according to the baseline percentiles. BMI z-score did not change during the 5 years, as body composition in terms of waist circumference, AMA and AFA.

Specifically, one patient who was underweight at baseline remained underweight, while the obese patient attained normal weight.

BMC and BMD increased significantly over time according to increasing age. FM% decreased significantly and gradually, in line with the increase in BW and the consequent growth in LM.

As expected, we found a negative trend in respiratory quotient going from indicative of a balanced diet (RQ > 0.80) to a hyperlipidic diet (RQ < 0.7), while fasting insulin and HOMA index decreased significantly after cKD initiation. No patient showed biochemical parameters above the cutoff; only two children reported a high HOMA-IR index value (> 3.16) and both TC and LDL-C levels above the cutoff, which returned to normalafter 1 year.

Overall, all biochemical parameters related to protein, lipid, glycaemic, and liver metabolism remained stable during the course of the dietary intervention.

## 4. Discussion

To our knowledge, this is the first study to investigate the long-term effects of cKD on nutritional status, in terms of anthropometric measurements, body composition, resting energy expenditure, and biochemical parameters, during a 5-year follow-up in a cohort of GLUT1-DS children and adolescents.

The reported negative RQ trend confirmed stable metabolic adaptation due to low carbohydrate intake and increased use of fat as an energy substrate and, thus, adherence to cKD (33, 34).

With regard to growth curves in children and adolescents, a slight yet non-significant decline in the z-scores of BMI-for-age, height-for-age, and weight-for-age can be observed, confirming the results reported by Tagliabue et al. who showed that after 6 months of KD, height, weight, and BMI remained approximately constant compared to pre-intervention values (14). Similarly, Ferraris et al. found that in 34 children with both GLUT1-DS and refractory epilepsy, 80% exhibited no growth retardation after 12 months of KD. Among all 15 children and adolescents in our study, only one female child remained underweight during the 5-year follow-up, and she was affected by both GLUT1-DS and cerebral palsy; the remaining children and adolescents maintained their growth trend or even improved their initial BMI z-score.

As for body composition, no significant changes in fat and lean mass standards were observed, probably due to the strict follow-up period and the closely monitored total daily calorie intake, which allows complete lipid oxidation without impairing body composition. A significant increase was found only in lean, bone mass, and not in body fat mass. Only in one female child who remained underweight and suffered from both GLUT1-DS and cerebral palsy did we observe a progressive reduction in lean mass, probably due to disease worsening. The remaining children all maintained their growth z-scores in both lean and body fat mass, except for the child who was obese at baseline. During the 5-year follow-up, in fact, he had an FM z-score in the normal range, in contrast to baseline where it was higher than normal range. These conflicting results might either be explained by reduced insulin leading to altered GH-pathways, or by the strictly controlled caloric intake, which reduces fat accumulation (35).

Our results also show that there was no significant worsening of bone health following a cKD, evidencing a significant increase in BMD as a function of increasing age. Bertoli et al. seem to support this hypothesis with a case series of adults affected by GLUT1-DS and other ultra-rare diseases (20, 21). In contrast, Bergqvist et al., in their 15-month longitudinal study of 25 children with refractory epilepsy, showed a decrease in BMC z-scores according to age and height (36). However, it should be considered that subjects already exhibited poor bone mineralization at pre-intervention, while in our study, patients had normal z-scores since pre-intervention (21). Moreover, in that study (36), patients were suffering from epilepsy, and therefore undergoing pharmacological treatments known to affect bone mineralization. In contrast, none of our patients were taking anti-epileptic drugs. The metabolic acidosis produced by cKD could play a role in the decrease in BMC, and studies in this regard with increased vitamin D and calcium supplementation or with alkalizing agents would be interesting (37). All of our patients take calcium and vitamin D supplements, as well as an alkalinizer, and this could be the other reason why we observed no worsening of bone. No studies have evaluated 24-h calciuria in GLUT1-DS patients, but our data suggest that chronic ketosis due to controlled cKD does not affect bone health. The role played by the Growt Hormone-stimulating hormone ghrelinmight also be of interest. However, in a previous study, we found no changes in ghrelin levels in 30 GLUT1-DS and refractory epilepsy patients on cKD, nor a correlation between cKDand nutritional status or body composition (16). In conclusion, these data suggest that a well-structured cKD provides adequate energy intake to maintain patients’ growth percentiles during childhood and adolescence (19), although GLUT1-DS disease has been found to be associated with nutrition and growth impairment at least until puberty (38). In addition to monitoring growth parameters, it is also recommended to periodically check body composition status in cKD subjects at risk for malnutrition (37).

Concerning biochemical parameters, no statistically significant changes were observed in lipid profile: only two children reported elevated TC and LDL-C levels above upper limits at baseline, which returned to normal as early as the first year of KD and remained in the normal range during the 5-year follow-up. These results are in contrast to those of the study by Reza Zamani et al. who reported increased TG, TC, and LDL, and decreased HDL in 33 children (39). In line with our results, a 10-year follow-up study by Heussinger et al. concluded that the lipid profile parameters reflected the pre-intervention status and showed no statistically significant changes in BMI, diastolic and systolic blood pressure, and carotid intima-media thickness as well (17), validating the hypothesis of fat utilization as an energy substrate that prevents body fat accumulation in children and adolescents. In addition, the absence of detrimental effects of fat intake on body fat accumulation and lipid profile could support the findings of Dehghan M et al. (40) that dietary fats, including saturated and unsaturated fatty acids, were associated with lower risk of total mortality and stroke when compared to high carbohydrate intake. Moreover, the quality of fats typical of Mediterranean countries (favoring foods rich in mono- and polyunsaturated fatty acids at the expense of saturated ones, such as oily fish, extra virgin olive oil, and nuts) has probably played a role in maintaining a stable lipid profile. On the other hand, insulin profile decreased significantly in the short term, as observed in short-term studies with follow-ups of 3, 6, or 12 months (2, 14, 16) and remained low even after 5 years. When a decrease in insulin is noted, it is reasonable to assume that this results in reduced stimulation of the GH hormone and, consequently, reduced IGF-1 production. Spulber et al. confirm this hypothesis by showing a negative correlation between β-hydroxybutyrate and growth rate and IGF-1 levels. Thus, even moderate calorie restriction could worsen growth rate in height, a hypothesis that should be considered when establishing a subject’s energy requirements (41). Constant monitoring of the REE and energy intake in line with measured needs allowed us to maintain physiological height growth. Regarding liver and kidney function, none of the patients reported abnormal values and significant changes in AST, ALT, GGT, urea, creatinine, and uric acid values.

The quality of the data collected is one of the strengths of our study, as all measurements and biochemical assays were mainly collected at the same center, thus ensuring less variability. It should also be noted that this is a multicenter study. In addition, it is the only study in the literature that has comprehensively assessed the nutritional status of GLUT-DS patients over such a long follow-up and has considered both body composition and REE, in addition to growth and biochemical parameters. REE and body composition were assessed by indirect calorimetry and DEXA, the gold standard methods for measuring energy needs and BMD, FM and LM, respectively. Finally, all of our patients were following the same diet, that is the cKD: although not traditionally recommended, patients with GLUT1-DS, especially during the adolescence, could be treated with a more moderate ketogenic, lower-fat diet, such as the Atkins diet, because it is hard to adhere to a strict cKD (5). It would be interesting to know the long-term effects on nutritional status of this other dietary treatment.

We are aware that there are a number of potential limitations: a control group was not included in the study, as GLUT1-DS management guidelines require the use of cKD from day 1 of diagnosis. Another limitation is the small number of patients recruited. However, it should be noted that GLUT1-DS is a very rare disease, so it is difficult to find a large representative sample: multicenter studies should be conducted to study larger samples. Furthermore, gender differences within the selected sample were not taken into account, with women representing the majority of the subjects included. It should also be considered that children with GLUT1-DS may have disease-related growth patterns. Therefore, an even more precise estimate of growth would require growth curves, which do not exist today, and should be designed *ad hoc* for this condition. As for BMD assessment, it should be noted that, although the subjects were adequately supplemented, no statistical analysis of changes in serum calcium and vitamin D values was performed. Finally, it is worth highlighting that the age of diagnosis and consequently the age of cKD initiation were different, which probably caused a different impact on nutritional status.

Here we present the 5-year effects of cKD. Long-term adherence to cKD had a good safety profile on anthropometric measurements, as well as body composition, resting energy expenditure, and biochemical parameters, and we found no evidence of potential adverse effects on the nutritional status of children and adolescents.

## Data availability statement

The raw data supporting the conclusions of this article will be made available by the authors, without undue reservation.

## Ethics statement

The studies involving human participants were reviewed and approved by Fondazione IRCCS Policlinico San Matteo di Pavia (reference number 20180083746). Written informed consent to participate in this study was provided by the participants’ legal guardian/next of kin.

## Author contributions

SB and RD: conceptualization and methodology. RD, AL, and AF: formal analysis. SB, RD, CL, MP, CF, AT, PV, VD, OS, and RP: investigation. RD, AF, SO, and RP: data curation. RD and MP: writing–original draft preparation. SB, AL, AB, CF, VD, AT, SO, PV and RP: writing–review and editing. SB: supervision. All authors contributed to the article and approved the submitted version.

## Funding

This research received ICANS internal funds.

## Conflict of interest

The authors declare that the research was conducted in the absence of any commercial or financial relationships that could be construed as a potential conflict of interest.

## Publisher’s note

All claims expressed in this article are solely those of the authors and do not necessarily represent those of their affiliated organizations, or those of the publisher, the editors and the reviewers. Any product that may be evaluated in this article, or claim that may be made by its manufacturer, is not guaranteed or endorsed by the publisher.
